# Relative Importance of Chemoautotrophy for Primary Production in a Light Exposed Marine Shallow Hydrothermal System

**DOI:** 10.3389/fmicb.2017.00702

**Published:** 2017-04-21

**Authors:** Gonzalo V. Gomez-Saez, Petra Pop Ristova, Stefan M. Sievert, Marcus Elvert, Kai-Uwe Hinrichs, Solveig I. Bühring

**Affiliations:** ^1^Hydrothermal Geomicrobiology Group, MARUM – Center for Marine Environmental Sciences, University of BremenBremen, Germany; ^2^Biology Department, Woods Hole Oceanographic Institution, Woods HoleMA, USA; ^3^Organic Geochemistry Group, MARUM – Center for Marine Environmental Sciences and Department of Geosciences, University of BremenBremen, Germany

**Keywords:** chemoautotrophy, marine shallow-water hydrothermal systems, lipid biomarker, stable isotope probing (SIP), fatty acids, Dominica (Lesser Antilles), *Zetaproteobacteria*, *Geothermobacter*

## Abstract

The unique geochemistry of marine shallow-water hydrothermal systems promotes the establishment of diverse microbial communities with a range of metabolic pathways. In contrast to deep-sea vents, shallow-water vents not only support chemosynthesis, but also phototrophic primary production due to the availability of light. However, comprehensive studies targeting the predominant biogeochemical processes are rare, and consequently a holistic understanding of the functioning of these ecosystems is currently lacking. To this end, we combined stable isotope probing of lipid biomarkers with an analysis of the bacterial communities to investigate if chemoautotrophy, in parallel to photoautotrophy, plays an important role in autotrophic carbon fixation and to identify the key players. The study was carried out at a marine shallow-water hydrothermal system located at 5 m water depth off Dominica Island (Lesser Antilles), characterized by up to 55°C warm hydrothermal fluids that contain high amounts of dissolved Fe^2+^. Analysis of the bacterial diversity revealed *Anaerolineae* of the *Chloroflexi* as the most abundant bacterial class. Furthermore, the presence of key players involved in iron cycling generally known from deep-sea hydrothermal vents (e.g., *Zetaproteobacteria* and *Geothermobacter*), supported the importance of iron-driven redox processes in this hydrothermal system. Uptake of ^13^C-bicarbonate into bacterial fatty acids under light and dark conditions revealed active photo- and chemoautotrophic communities, with chemoautotrophy accounting for up to 65% of the observed autotrophic carbon fixation. Relatively increased ^13^C-incorporation in the dark allowed the classification of *ai*C_15:0_, C_15:0_, and *i*C_16:0_ as potential lipid biomarkers for bacterial chemoautotrophy in this ecosystem. Highest total ^13^C-incorporation into fatty acids took place at the sediment surface, but chemosynthesis was found to be active down to 8 cm sediment depth. In conclusion, this study highlights the relative importance of chemoautotrophy compared to photoautotrophy in a shallow-water hydrothermal system, emphasizing chemosynthesis as a prominent process for biomass production in marine coastal environments influenced by hydrothermalism.

## Introduction

The discovery of deep-sea hydrothermal vents on the Galápagos Rift in 1977 identified for the first time a marine ecosystem where chemosynthesis, as opposed to photosynthesis, was the predominant form of organic carbon production (Corliss et al., 1979; Jannasch and Wirsen, 1979; Jannasch and Mottl, 1985). Chemoautotrophic microorganisms in hydrothermal systems are able to assimilate inorganic carbon into biomass and effectively transfer the energy from the geothermal source to higher trophic levels (e.g., Sievert and Vetriani, 2012). The general knowledge of chemoautotrophy at deep-sea hydrothermal vents has advanced considerably over the last years (e.g., Flores et al., 2011; Hügler and Sievert, 2011; Dahle et al., 2015; Stokke et al., 2015; Fortunato and Huber, 2016; McNichol et al., 2016). However, hydrothermal systems can harbor heterogeneous microbial habitats (e.g., Santelli et al., 2008; Flores et al., 2011; Olins et al., 2013; Reeves et al., 2014; Stokke et al., 2015) and it is often difficult to obtain samples or to perform *in situ* measurements at deep-sea vents (e.g., Sievert and Vetriani, 2012; Reeves et al., 2014; McNichol et al., 2016). Thus, there are still significant gaps in relation to the microbial biogeochemistry of hydrothermal systems in determining the function of different community members and the relevance of the metabolic pathways carried out by them (e.g., Sievert and Vetriani, 2012; Reeves et al., 2014).

Hydrothermal systems occur over a wide depth range in the oceans, from the intertidal to the abyss (e.g., Tarasov et al., 2005; Hawkes et al., 2014). Marine shallow-water hydrothermal systems (<200 m water depth) are relatively easily accessible extreme environments with unique biogeochemical conditions (Tarasov et al., 2005). Energy sources for primary production in these systems become available when the hot, reduced hydrothermal fluids mix with the cold, oxygenated seawater (e.g., Amend and Shock, 1998). In contrast to deep-sea vents, shallow-water vents not only support chemosynthetic processes, but also primary production by photosynthesis due to the availability of light (Tarasov et al., 2005). Accordingly, shallow-water systems are generally characterized by a higher input of autochthonous organic matter compared to deep-sea vents, where new biomass is thought to be exclusively produced by chemosynthesis (Jannasch and Mottl, 1985). Furthermore, the additional input of allochthonous organic matter generated on land, in the vicinity of the vents, or in the water column above the vents may sustain heterotrophic processes (e.g., Sievert et al., 2000). Marine shallow-water hydrothermal systems have been investigated using geochemical approaches (e.g., Dando et al., 1999; McCarthy et al., 2005; Tarasov et al., 2005 and references therein; Price et al., 2013; Gomez-Saez et al., 2016 Yücel et al., 2013) and bacterial community structure analyses (e.g., Gugliandolo and Maugeri, 1998; Sievert et al., 1999, 2000; Giovannelli et al., 2013; Meyer-Dombard et al., 2013). However, comprehensive studies targeting the predominant biogeochemical processes at shallow-water hydrothermal systems are rare, and a holistic understanding of the functioning of these ecosystems is currently lacking.

Several isotope-based methods have been introduced in recent years for cultivation-independent characterization of active microorganisms in environmental samples (e.g., Hesselsoe et al., 2005; Dyksma et al., 2016; Fortunato and Huber, 2016). The analysis of lipid signatures in natural environments offers a unique approach, as they provide quantitative information about the community structure without the necessity of culturing, as well as revealing information about the adaptation of microbes to varying environmental conditions (e.g., White, 1988; Hayes et al., 1990; Hinrichs et al., 1999; Hayes, 2001; Lincoln et al., 2014). In hydrothermal environments, lipids have been used to decipher carbon flow at deep-sea vents (e.g., Bradley et al., 2009; Reeves et al., 2014) and in terrestrial hot springs (e.g., van der Meer et al., 2000; Schubotz et al., 2013, 2015). Approaches utilizing substrates labeled with stable isotopes such as ^13^C in combination with mass spectrometric determination of the labeled fatty acids have been furthermore widely used for the detection and quantitative assessment of physiologically active bacteria in complex microbial communities (e.g., Pel et al., 1997; Boschker et al., 1998, 2014; Hanson et al., 1999; Nold et al., 1999; Bull et al., 2000; Knief et al., 2003; Kellermann et al., 2012; Wegener et al., 2012, 2016; Bühring et al., 2014). Recent studies concluded that dark carbon fixation can be a major process in coastal sediments not influenced by hydrothermal activity, representing nearly half of global chemoautotrophy in the ocean and being predominantly performed by *Gammaproteobacteria* (Middelburg, 2011; Boschker et al., 2014; Dyksma et al., 2016). However, the analysis of lipid signatures has not yet been applied to elucidate the relative importance of chemosynthesis for primary production in marine shallow-water hydrothermal systems.

**FIGURE 1:**
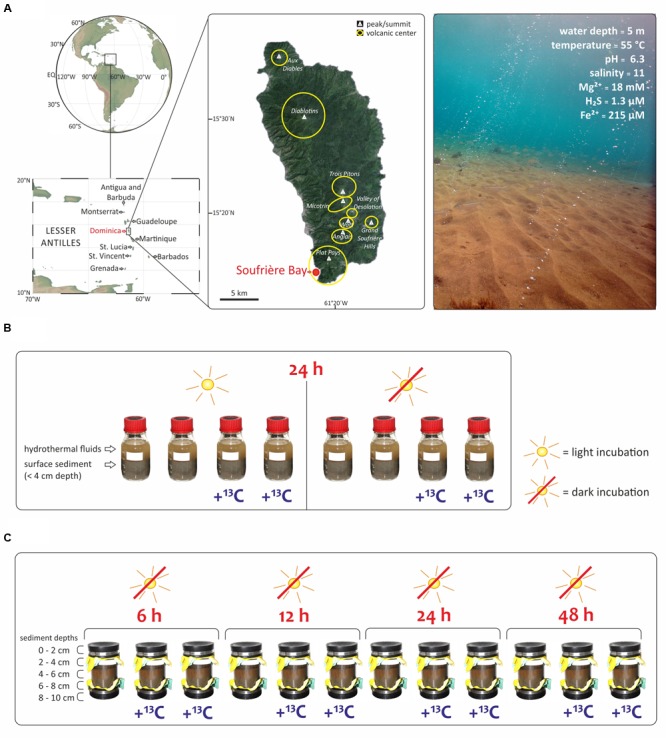
**Study area and experimental set up of this study. (A)** The shallow hydrothermal system is located in Soufrière Bay in the southwest of Dominica (Lesser Antilles) adapted from Gomez-Saez et al. (2015). Maps were created using Ocean Data View (R. Schlitzer, http://odv.awi.de) and Google Earth (http://earth.google.com). Submarine photo courtesy of A. Madisetti showing light reaching the surface sediment at 5 m water depth and visible orange color due to Fe^3+^ oxides precipitates. Geochemistry values taken from Gomez-Saez et al. (2015, 2016). **(B)** SIP-experiment to evaluate the effect of light with incubations under light and dark conditions. Bottles of 250 mL were filled with hydrothermal fluids and surface sediment (<4 cm depth) and incubated during 24 h. **(C)** SIP-experiment to investigate different redox interfaces under dark conditions as a function of incubation time (6 to 48 h) and sediment depth at five intervals (0–2, 2–4, 4–6, 6–8, and 8–10 cm). All samples in **(B,C)** were incubated at 55°C, corresponding to the *in situ* temperature of the hydrothermal fluids at the sampling site.

**FIGURE 2:**
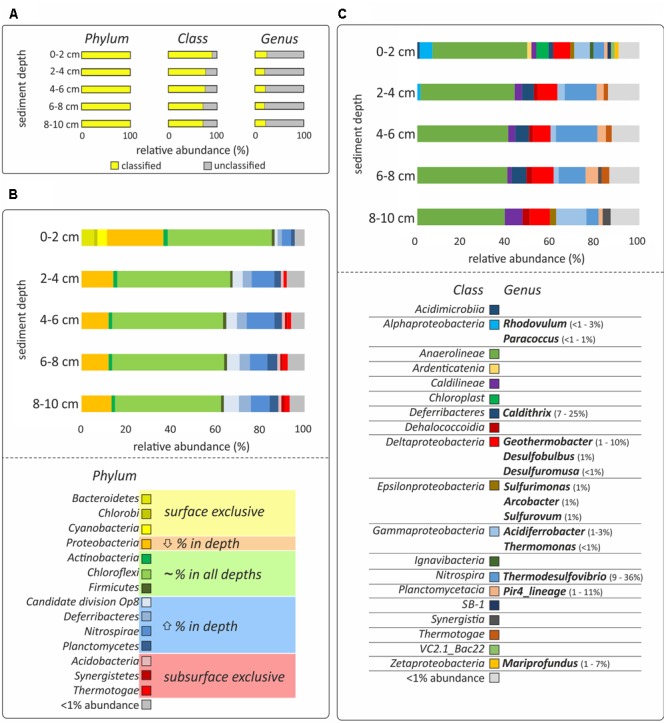
**DNA-based analysis of bacterial diversity. (A)** The percentage of classified and unclassified phyla, classes and genera. **(B,C)** Relative sequences abundances at the phylum and class level, respectively. Only bacteria with more than 1% of relative abundance are shown and the hydrothermal sediments were incubated for 48 h, which might bias the results. Classes with less than 1% presence are grouped in the category “<1% abundance.” All relative abundances in **(B,C)** are normalized to 100%, considering the classified sequences presented in yellow in **(A)**. Color code in **(B)** indicates if the phylum was only present in surface sediment (0–2 cm; yellow), decreased its relative abundance with depth (orange), did not show relevant differences of ±5% of relative abundance in the different layers (green), increased the relative abundance with depth (blue) or was exclusive to the subsurface layers (2–10 cm; red). Genera identified as the most characteristic of the given taxonomical class and their relative abundance are presented in **(C)**.

The aim of the present study was to investigate the relative contribution of chemoautotrophy for total microbial carbon fixation in a light-exposed, iron-enriched marine shallow-water hydrothermal system, where the continuous supply of reduced substances from below may support chemoautotrophy, while the presence of light sustains photoautotrophy. We explored a marine shallow-water hydrothermal system located at 5 m water depth in Soufrière Bay on the southwest coast of Dominica (Lesser Antilles) (**Figure 1A**). Dominica belongs to the Lesser Antilles islands, which represents one of only two active arc systems in the Atlantic Ocean. Although most of these islands have a single volcanic center (e.g., Saba, Statia, Nevis, Montserrat, Guadeloupe, and Saint Vincent), Dominica has nine potentially active volcanic centers (Lindsay et al., 2005; Joseph et al., 2011). Therefore, Dominica has been the most volcanically active island in the Lesser Antilles arc over the last 100,000 years and one of the most active worldwide (Wadge, 1984; Lindsay et al., 2005). Submarine hydrothermal venting off Dominica occurs mainly along the submerged flank of the Plat Pays Volcanic Complex in the south-west of the island, with fluid temperatures ranging between 44 and 75°C (McCarthy et al., 2005; Gomez-Saez et al., 2015, 2016; Kleint et al., 2015). Applying an integrated approach, we combined stable isotope probing (SIP) of lipid biomarkers with DNA-based analysis of bacterial diversity, and quantified the uptake of ^13^C-bicarbonate into lipid biomarkers (1) under light and dark conditions (**Figure 1B**), and (2) at different redox interfaces under dark conditions as a function of incubation time and sediment depth (**Figure 1C**).

## Materials and Methods

### Field Sampling and ^13^C-labeling Experiments

The hydrothermal fluid, seawater, and sediment samples for this study were taken during a field expedition to Dominica (**Figure 1A**) in April 2013. Identification of locations where hot fluids percolate through the sediment was carried out by SCUBA diving using previously described *in situ* temperature probes (e.g., Price et al., 2013; Kleint et al., 2015). The pH and salinity were measured *in situ* at the point of fluid discharge from the sediments, using a WTW pH meter 3210 with Mic-D electrode. Fluid samples for geochemical analyses were collected with a funnel at the point of discharge out of the sediments, channeling the fluid into a food-grade large volume nylon bag as described previously (Gomez-Saez et al., 2015). Polycarbonate core liners (20 cm long) with rubber end caps (**Figure 1C**) were used for sampling sediment and overlying water, and transported back to the laboratory within 2 h after sampling in an upright position to ensure minimum disturbance of the sediment. The sediment from the cores was sliced at five different depths (0–2, 2–4, 4–6, 6–8, and 8–10 cm). Two SIP-experiments were carried out immediately upon arrival in the laboratory. The first SIP-experiment evaluated the effect of light (**Figure 1B**). Accordingly, eight parallel incubations of Soufriére vent surface sediment from the first 4 cm were used. Firstly, 250 mL pre-combusted glass bottles were filled with 150 mL sediment and mixed with 100 mL of hydrothermal fluids from Soufrière vents. The eight closed bottles were incubated for 24 h at 55°C, corresponding to the *in situ* temperature. Half of the samples were incubated in the dark, covered with aluminum foil, while the other half were exposed to light (**Figure 1B**). Furthermore, half of the samples were incubated with ^13^C-bicarbonate addition to set a final concentration of 6.5 mM (25 mL of NaH^13^CO_3_ solution, ^13^C 99%; Cambridge Isotope Laboratories, Inc.) and the other half of the samples were incubated without tracer addition (**Figure 1B**). The second SIP-experiment investigated different redox interfaces under dark conditions. Twelve sediment cores were sampled from the same venting area and a mixture of ^13^C-bicarbonate and hydrothermal fluid was injected into eight sediment cores at five different depth layers (0–2, 2–4, 4–6, 6–8, and 8–10 cm) to set a final concentration of 6.5 mM. The cores were subsequently incubated in the dark at 55°C together with four cores that did not receive a tracer addition. At each sampling time (6, 12, 24, and 48 h), two cores with and one core without label addition were subsampled at 2 cm depth intervals (**Figure 1C**). Each sediment slice was transferred into 150 mL pre-cleaned vials (Carl Roth, Germany), which were kept frozen at -20°C until lipid extraction conducted in the laboratory in Bremen, Germany.

### Bacterial Diversity Analysis

The bacterial diversity of the five sediment depth layers (0–2, 2–4, 4–6, 6–8, and 8–10 cm) was analyzed from one of the cores incubated in the dark for 48 h, which might bias the results if interpreted as natural community composition. DNA was extracted from 0.5 g of sediment using the FastDNA^®^ SPIN Kit for Soil (MP Biomedicals, Irvine, CA, USA), and finally eluted in 50 μL 1x Tris-EDTA buffer (Promega, Madison, WI, USA). Bacterial communities were analyzed by sequencing the v3 – v4 hypervariable region of the 16S rRNA gene using the primer pair S-D-Bact-0341-b-S-17 and S-D-Bact-0785-a-A-21, with Illumina MiSeq at MR DNA (Shallowater, TX, USA). Multifasta files were parsed, checked for quality and trimmed with split_libraries.py command as implemented in QIIME v1.9.1 (Caporaso et al., 2010). Sequences processing, including alignment, quality control, dereplication, clustering and classification, was done with the SILVAngs analysis pipeline 1.2 (SILVA SSU Ref dataset 119.1; Quast et al., 2013). Sequences were clustered in operational taxonomic units (OTU_0.03_) based on 97% sequence similarity. All downstream statistical analyses were done in R (R Core Team, 2014), using the vegan package (Oksanen et al., 2015) and custom-based scripts. All analyses were done excluding OTU_0.03_ singletons, i.e., OTU_0.03_ represented by only one sequence in the whole dataset. Prior to this, the data were normalized to the sample with the least number of sequences (8–10 cm depth; 28,305 sequences). Data interpretation was based on the relative abundances of the sequences classified at the class or genus level. The percentage of unclassified sequences was 10% for the surface sample (0–2 cm) and 26 ± 3% for the subsurface samples (2–10 cm) at the class level and 79 ± 2% at the genus level in all sediment depths (**Figure 2A**). Sequence data from this study were submitted to NCBI SRA (BioProject ID: PRJNA379939).

### Lipid Biomarkers Analysis

Total lipids were extracted from freeze-dried sediment samples following a protocol based on Bligh and Dyer (1959) and modified by Sturt et al. (2004). This method consists of four steps using dichloromethane/methanol twice with each phosphate and trichloroacetic acid buffers. 2-methyl-octadecanoic acid was used as internal standard and added prior to extraction. An aliquot of the total lipid extract was saponified following Elvert et al. (2003). This method includes a base saponification using potassium hydroxide in methanol, base extraction of the neutral lipids and acid extraction of the free fatty acids. Prior to analysis, fatty acids were derivatized using boron trifluoride (BF_3_) in methanol (Merck), leading to fatty acid methylesters. Identification of fatty acids was performed by gas chromatography – mass spectrometry (GC-MS) combining an Agilent 6890N gas chromatograph with an Agilent 5973N mass selective detector. The capillary column was Restek Rtx^®^-5MS silica column with a length of 30 m, an internal diameter of 0.25 mm, and a film thickness of 0.25 μm. The operating conditions of the GC were as follows: 2 μL sample volume were injected for 1 min. Temperature was increased from 60 to 150°C at 10°C min^-1^, then to 320°C at 4°C min^-1^. The total running time was 60 min. Helium was used as carrier gas with a flow-rate of 1.0 mL min^-1^. The electron impact mass spectra were recorded at a range of 50–700 m/z. Fatty acids were quantified by gas chromatography coupled to a flame ionization detector (GC-FID) using the same oven operating conditions as for the GC – MS. The carbon isotopic compositions were determined by GC-isotope ratio-MS (GC-irMS) using a Thermo Scientific Trace GC Ultra coupled to a Thermo Scientific Delta V Plus irMS and the same operating conditions described before. The reference gas was CO_2_ and squalane was used as injection standard to check for internal precision. The carbon isotope ratios were expressed in the delta notation (δ^13^C) relative to Vienna Peedee Belemnite (^13^C/^12^C_V PDB_ = *R*_V PDB_ = 0.0112372) according to δ^13^C (‰) = [(*R*_sample_ /*R*_std_)-1] × 1000, where *R*_sample_ and *R*_std_ are the ^13^C/^12^C ratio values of sample and standard, respectively. Incorporation of ^13^C in the SIP experiments is reflected as an excess compared to the amount of ^13^C in background samples and is expressed in terms of total uptake as described by Middelburg et al. (2000). Total uptake of ^13^C was calculated as the product of excess ^13^C (E) and concentration of the respective compound. E was the difference between the fraction F of the sample and background:

$$\begin{array}{c} E=\;F_{\mathrm{sample}}-F_{\mathrm{background}},\mathrm{where}F\;={\;}^{13}C/{(}^{13}{C+}^{12}C)\\ \;=\;R/(R+1)\;\mathrm{and}\;R\;=\;(\delta^{13}C/1000+1)\;\times \;R_{\mathrm{VPDB}}. \end{array}$$

### Statistical Analysis

A non-metric multidimensional scaling (NMDS) analysis was performed in order to assess how incubated samples (during 6–48 h in the dark) were similar or differ from each other based on the incorporation of ^13^C-bicarbonate into different fatty acids. Dissimilarity among samples was calculated based on the Bray-Curtis dissimilarity index. Separation of groups identified with the NMDS analysis was furthermore tested for significance using the non-parametric Analysis of Similarity Test (ANOSIM). Correlation analyses based on Pearson’s correlation coefficient were performed in order to test if incorporation of ^13^C-bicarbonate into the same fatty acids was significantly correlated with sediment depth.

## Results

### Bacterial Community Composition

Bacterial community analysis of the incubated samples revealed variations in the taxonomical composition as a function of sediment depth. The most pronounced differences were observed between surface (0–2 cm) and subsurface (2–10 cm) layers, although the removal of the natural environment and subsequent 48 h incubation prior to analysis might have an impact on the abundance of the different bacterial populations identified (**Figure 2B**). *Anaerolineae* of the *Chloroflexi* was the most abundant bacterial class, showing a similar relative abundance at all depth layers (28–38%, **Figure 2**). Other *Chloroflexi* present in more than 1% of relative abundance were *Caldilineae*, increasing in relative abundance with depth (2–6%), *Ardenticatenia*, present only at the sediment surface (<2%), and *Dehalococcoidia*, present only at the sediment subsurface (1–2%, **Figure 2C**). On the other hand, *Bacteroidetes* (6%), *Cyanobacteria* (4%), and *Chlorobi* (2%), were found almost exclusively at the sediment surface, while *Acidobacteria* (1%), *Synergistetes* (1%), and *Thermotogae* (1–2%), were mainly present in subsurface layers (2–10 cm) (**Figure 2C**). The phyla that increased in relative abundance with depth were candidate division OP8 (1–7%), *Deferribacteres* (2–5%), *Nitrospirae* (4–12%), and *Planctomycetes* (2–5%, **Figure 2B**). These phyla also comprised the most dominant genera of the whole dataset: *Thermodesulfovibrio* (*Nitrospira*; 9–36%), *Caldithrix* (*Deferribacterales*; 7–25%), and Pir4_lineage (*Planctomycetes*; 1–11%) (**Figure 2C**). In contrast, *Proteobacteria* was the only phylum for which the relative abundance decreased with depth (25–12%, **Figure 2B**).

The abundance of potential iron-oxidizers was highest in the surface layer. These include the phototrophic *Rhodovulum* (<1–3%), and the chemoheterotrophic *Paracoccus* (<1–1%), belonging to the *Alphaproteobacteria*, and *Mariprofundus* (1–7%), belonging to the *Zetaproteobacteria* (**Figure 2C**). A similar distribution was revealed for *Epsilonproteobacteria* (2%), which included sequences affiliated to numerous potentially chemolithoautotrophic bacteria, such as *Sulfurimonas* (1%), *Arcobacter* (1%), and *Sulfurovum* (1%) (**Figure 2C**). In contrast, *Deltaproteobacteria* (6–7%), and *Gammaproteobacteria* (2–10%), were found at all depths (**Figure 2C**). The most abundant sequences of these classes were affiliated to numerous potentially iron-utilizing and/or chemolithoautotrophic genera, such as *Acidiferrobacter* (1–3%), *Thermomonas* (≤1%), *Geothermobacter* (1–10%), *Desulfobulbus* (1%), and *Desulfuromusa* (≤1%) (**Figure 2C**).

### ^13^C-bicarbonate Incubations

**FIGURE 3:**
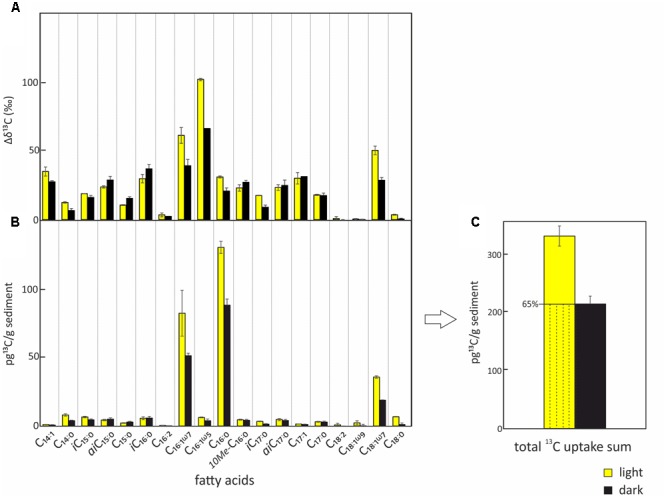
**Uptake of ^13^C-bicarbonate into lipid biomarkers under light and dark conditions.** Total uptake of ^13^C-bicarbonate into fatty acids after incubations under light (yellow) and dark (black) conditions including standard deviations of repeated measurements. **(A)** Relative change of δ^13^C in the different bacterial fatty acids over the course of the experiment. **(B)** Absolute ^13^C-bicarbonate uptake into the different bacterial fatty acids. **(C)** Total quantified ^13^C-incorporation in fatty acids. The total uptake under light conditions which is attributed to chemoautotrophy is indicated by the dashed area.

**FIGURE 4:**
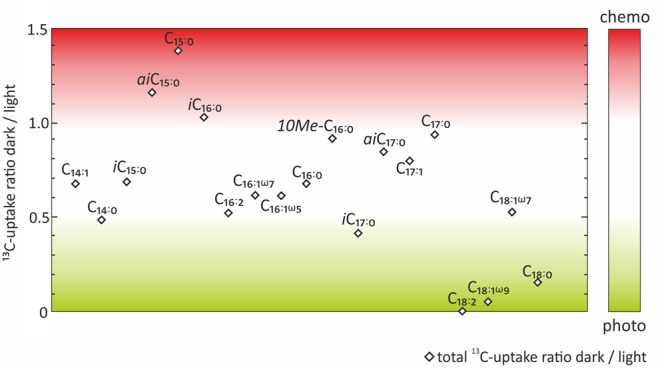
**Ratios between dark and light incubations of the absolute ^13^C-uptake into fatty acids.** Color code indicates which fatty acids were more likely to be indicative of chemoautotrophy (red) or photoautotrophy (green).

**FIGURE 5:**
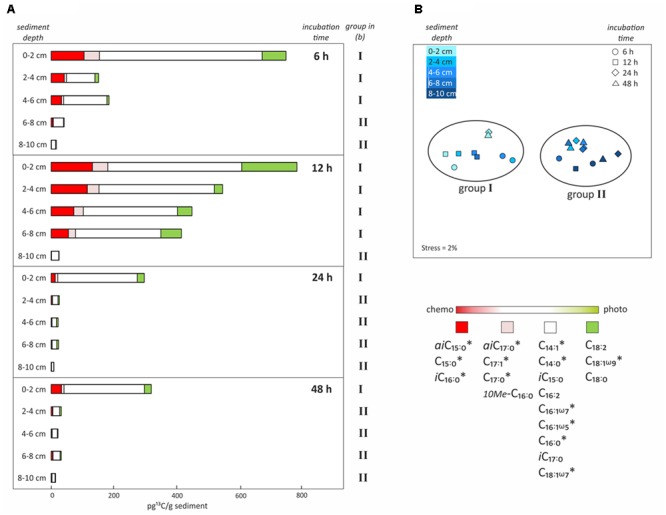
**Uptake of ^13^C-bicarbonate into lipid biomarkers under dark conditions as a function of incubation time and sediment depth. (A)** Total uptake of ^13^C-bicarbonate into different fatty acids after sediment core incubations under dark conditions as a function of time (6, 12, 24, or 48 h) and sediment depth intervals (0–2, 2–4, 4–6, 6–8, or 8–10 cm). Colors reflect the likely source of the fatty acids as inferred from the dark/light ratios of ^13^C-bicarbonate uptake depicted in **Figure 4**. **(B)** Non-metric multidimensional scaling analysis (NMDS) based on total ^13^C uptake into each fatty acid in all depths and of incubation times, identifying the presence of two groups of samples that are statistically different from each other. ^∗^Indicates significant negative correlation between fatty acid and sediment depth.

The two SIP-experiments revealed uptake of ^13^C-bicarbonate under light and dark conditions suggesting potentially active photo- and chemoautotrophic communities in the Dominica shallow-water hydrothermal system (**Table 1** and **Figures 3**–**5**). The likely natural isotopic composition of fatty acids was inferred from incubations without tracer addition, averaging – 29.0 ± 2.6‰ in the light and – 29.2 ± 3.8‰ under dark conditions (**Table 1**), which is suggestive of the use of the Calvin-Benson-Basham cycle for carbon fixation (Hügler and Sievert, 2011). Highest ^13^C-enrichment was found for the monounsaturated fatty acids C_16:1ω5_ (58.9‰), C_16:1ω7_ (25.3‰), and C_18:1ω7_ (16.3‰) during light incubation, and C_16:1ω5_ (22.5‰), C_16:1ω7_ (6.6‰) and the branched fatty acid *i*C_16:0_ (5.6‰) during dark incubation (**Table 1**). Total uptake of ^13^C-bicarbonate into fatty acids accounted for up to 329 ± 34 pg ^13^C g^-1^ sediment (dry weight) in the light and 213 ± 27 pg ^13^C g^-1^ of sediment (dw) in the dark (**Figures 3B,C**), accounting for incorporation rates of 14 and 9 pg ^13^C g^-1^h^-1^, respectively. As photoautotrophy can be ruled out in the dark, we estimated that chemoautotrophy accounted for up to 65% of the total autotrophic carbon fixation in fatty acids compared to combined photo- and chemoautotrophy during light incubation (dashed lines; **Figure 3C**).

**Table 1:** Relative distribution and changes in δ^13^C values of fatty acids ranging from C_14_ to C_18_ during light and dark incubations with and without addition of ^13^C-bicarbonate.

Fatty acid	Light incubation			Dark incubation		
	%	w/o bicarbonate (‰)	w ^13^C bicarbonate (‰)	%	w/o bicarbonate (‰)	w ^13^C bicarbonate (‰)
C_14:1_	0.2	–33.2	–1.5	0.1	–36.0	–11.0
C_14:0_	3.8	–27.9	–16.6	3.6	–28.9	–22.9
*i*C_15:0_	2.1	–27.0	–10.0	1.8	–27.1	–12.4
*ai*C_15:0_	1.1	–28.3	–6.7	1.1	–29.8	–3.7
C_15:0_	1.2	–27.0	–17.4	1.2	–32.9	–18.8
*i*C_16:0_	1.1	–27.2	–0.2	1.0	–27.9	5.6
C_16:2_	0.6	–33.9	–30.7	0.5	–32.6	–30.4
C_16:1ω7_	7.8	–30.2	25.3	7.9	–28.9	6.6
C_16:1ω5_	0.4	–33.5	58.9	0.4	–37.6	22.5
C_16:0_	24.8	–28.7	–0.6	26.0	–28.2	–9.3
*10Me-*C_16:0_	1.2	–30.0	–9.1	1.0	–28.6	–4.0
*i*C_17:0_	1.1	–29.9	–14.0	1.0	–23.3	–15.1
*ai*C_17:0_	1.2	–29.1	–7.9	1.0	–27.8	–5.3
C_17:1_	0.3	–30.6	–3.3	0.2	–32.4	–4.0
C_17:0_	1.0	–26.1	–9.7	1.0	–24.8	–8.8
C_18:2_	5.2	–24.3	–23.6	4.5	–24.5	–24.5
C_18:1ω9_	31.9	–27.2	–26.8	31.0	–26.7	–26.7
C_18:1ω7_	4.1	–29.5	16.2	3.9	–30.0	–3.9
C_18:0_	10.9	–27.4	–24.2	12.6	–26.6	–26.2

In order to evaluate the potential of specific fatty acids as biomarkers for either chemo- or photoautotrophic processes, we calculated the ratios between dark and light incubations (**Figure 4**) of the absolute ^13^C uptake (**Figure 3B**). Those fatty acids showing a dark/light ratio ≥ 1 indicate a higher ^13^C-uptake under dark than under light conditions, and were categorized as most characteristic chemoautotrophic biomarkers. In contrast, fatty acids with dark/light ratio values of 0 would be indicative of null incorporation during dark incubation and therefore strongly linked to photoautotrophy. Into this category, we also included those fatty acids with at least five times more ^13^C-uptake under light than under dark conditions, i.e., dark/light ratios < 0.2 (**Figure 4**). Incorporation of ^13^C into bacterial fatty acids differed as a function of light availability, supporting the classification of fatty acids being dominantly produced during chemo- or photoautotrophy in shallow-water hydrothermal systems. Fatty acids with higher ^13^C incorporation under dark conditions (ratio ≥ 1) were identified as potential chemoautotrophic biomarkers in function of their absolute ^13^C uptake ratios, specifically including *ai*C_15:0_ (1.16), C_15:0_ (1.38), and *i*C_16:0_ (1.03) (**Figure 4**). In addition, fatty acids that were linked to chemoautotrophy included the fatty acids *10Me-*C_16:0_ (0.92), *ai*C_17:0_ (0.85), C_17:1_ (0.80) and C_17:0_ (0.94) because their dark/light ratios were found to be between 0.8 and 1. The most dominant fatty acids in our study were C_16:0_, C_16:1ω7_ and C_18:1ω7_ with highest absolute ^13^C-incorporation, but they could not be classified in terms of chemoautotrophy or photoautotrophy as their ratio dark/light was between 0.5 and 0.7 (**Figure 4**). In contrast, the fatty acids categorized as being most likely linked to photoautotrophs were C_18:2_ (0), and C_18:1ω9_ (0.06), revealing ^13^C-incorporation from the added tracer only under light conditions, as well as C_18:0_ (0.16), for which the uptake under light conditions was very low, but at least five times higher than under dark conditions (ratio < 0.2) (**Table 1** and **Figure 4**).

Incubations in the dark as a function of time (6, 12, 24, or 48 h) and sediment depth (0–2, 2–4, 4–6, 6–8, or 8–10 cm) were performed to obtain further insights into the chemosynthetic activity at different redox interfaces. Highest total ^13^C-incorporation was always found at the sediment surface (0–2 cm), where it decreased by ∼50% after 12 h, being 763 and 798 pg ^13^C g^-1^ sediment at 6 h and 12 h, then 302 and 325 pg ^13^C g^-1^ sediment at 24 and 48 h, respectively. This resulted in a decreasing rate of tracer uptake per hour from 127, to 67, to 13 and finally to 7 pg ^13^C g^-1^h^-1^ (0–2 cm; **Figure 5A**). The decrease in ^13^C-incorporation observed after longer incubation was even more evident in deeper layers (2–8 cm), resulting in rates < 30 pg ^13^C g^-1^h^-1^ after 6 h, < 50 pg ^13^C g^-1^h^-1^ after 12 h and only < 1 pg ^13^C g^-1^h^-1^ after 24 and 48 h (**Figure 5A**). The deepest layer (8–10 cm) presented low, but consistent ^13^C-incorporation of 9 to 26 pg ^13^C g^-1^ sediment (dw) for all incubation times (rates ∼1 pg ^13^C g^-1^h^-1^) (**Figure 5B**). NMDS analysis based on the total uptake of ^13^C into every fatty acid for all depths and incubation times, revealed two groups of samples that were statistically and significantly different from each other (ANOSIM *R* value = 0.9; Bonferroni corrected *p*-value = 0.0001; **Figure 5B**). One group included all surface samples together with subsurface samples down to 6 and 8 cm depth from 6 to 12 h incubations, respectively (group I; **Figure 5B**). The second group included all subsurface samples that were incubated for 24 and 48 h (2–10 cm), as well as the deepest layers (6–10 cm and 8–10 cm) incubated for 6 and 12 h (group II; **Figure 5B**). Furthermore, incorporation of ^13^C into most of the bacterial fatty acids was significantly negatively correlated with sediment depth (*r*^2^ = -0.5 to -0.7, *p*-value < 0.005; Pearson correlation; **Figure 5B**).

## Discussion

### Imprint of Vent Geochemistry onto Sedimentary Microbial Diversity

We explored a shallow-water hydrothermal vent system located at 5 m water depth in Soufrière Bay in the southwest of Dominica (Lesser Antilles) (**Figure 1A**). Hydrothermal fluids of the studied vent system had a temperature of 55°C and a slightly acidic pH of 6.3, in contrast to the ambient seawater with a temperature of 28°C and a pH of 7.9 (Gomez-Saez et al., 2015). Soufrière Bay hydrothermal fluids seem to be composed mainly of meteoric water as indicated by the salinity and the concentrations of major elements (e.g., salinity 11; Mg^2+^ = 18 mM; Gomez-Saez et al., 2015). Soufrière Bay hydrothermal vent fluids contained high amounts of ferrous iron (Fe^2+^ 215 μM; Gomez-Saez et al., 2015), which gets oxidized upon contact with oxygenated seawater, forming orange patches of hydrous ferric (Fe^3+^) oxide precipitates on the sediment surface (McCarthy et al., 2005; Gomez-Saez et al., 2015; **Figure 1A**). Therefore, the geochemistry of the Dominica shallow hydrothermal system is characterized by iron redox cycling, in line with the presence of diverse and abundant key players known to be involved in iron cycling (**Figure 2**). This suggests that microbially mediated iron cycling plays an important role in the biogeochemistry of the Dominica shallow hydrothermal system, which would be in accordance with other iron-enriched shallow-water hydrothermal systems off Santorini (Greece) or Tutum Bay (Papua New Guinea) (Handley et al., 2010; Meyer-Dombard et al., 2013).

In marine coastal sediments without hydrothermal activity, *Gammaproteobacteria* have been reported to account for 70–86% of dark carbon fixation (Dyksma et al., 2016). In our study, we detected a high diversity of *Bacteria* known to be involved in iron-oxidation, belonging mainly to the *Alpha-, Gamma-*, and *Zetaproteobacteria* (**Figure 2**). The most well documented marine iron oxidizer is *Mariprofundus ferrooxydans* belonging to the *Zetaproteobacteria* (Emerson et al., 2007, 2010). *Mariprofundus* was among the ten most abundant genera in our dataset (**Figure 2**). Sequences belonging to this genus were mainly identified in the surface layer, where physicochemical conditions were most suitable for iron-oxidation due to the simultaneous presence of both dissolved Fe^2+^ and oxygen. This is in accordance with the cultivation conditions of *Mariprofundus*, which grows as an oxygen-dependent obligate lithotroph at a pH range of 5.5–7.2 (Emerson et al., 2007, 2010). Iron-oxidizing *Zetaproteobacteria* have previously been found mainly at deep-sea hydrothermal vents (Emerson and Moyer, 2002; Kato et al., 2009; Emerson et al., 2010; McAllister et al., 2011 and references therein); but also in brackish environments (McBeth et al., 2011), in a groundwater laboratory under the Baltic Sea (Ionescu et al., 2015), as well as at marine shallow-water hydrothermal systems off Santorini (Greece) (Hanert, 2002; Handley et al., 2010). In our study, we detected *Mariprofundus* at a temperature of 55°C, which is in the range of Santorini hydrothermal sediments < 40°C (Handley et al., 2010) and the high temperatures of > 90°C detected at the vent source of Tutum Bay (Papua New Guinea), where *Zetaproteobacteria* in surface sediment were identified (Meyer-Dombard et al., 2013). Other highly abundant chemolithotrophs that potentially obtain energy via oxidation of iron were detected at the Dominica vent, and included bacteria affiliated with *Acidiferrobacter*, a genus that is distantly related to the well-known thermotolerant (maximum growth temperature 47°C) iron-oxidizing genus *Acidithiobacillus* (Hallberg et al., 2010).

In addition to iron-oxidizers, we could also identify numerous taxa potentially capable of reducing iron, mainly *Deltaproteobacteria*. Iron reduction metabolism is difficult to be inferred from phylogeny because many of these microorganisms are capable of using electron acceptors other than Fe^3+^ (e.g., Handley et al., 2010; Ionescu et al., 2015). Although *Shewanellaceae* of the *Gammaproteobacteria* are among the most commonly identified iron-reducing bacteria (Zhang et al., 2003), they were hardly detected in this study. In contrast, the thermophilic genus *Geothermobacter*, originally isolated from a deep-sea hydrothermal vent (Kashefi et al., 2003), was by far the most dominant deltaproteobacterium that could be identified as a potential iron-reducer. *Geothermobacter* was among the five most abundant genera of the whole dataset, indicating its importance for iron cycling at the studied vent system. We further detected other less abundant iron-reducing taxa that are also known to be able to use sulfur as electron acceptor, like *Deferribacteres, Desulfobulbus*, and *Desulfuromonas*. This is in accordance with previous studies of hydrothermal ecosystems, including shallow-water vents (Takai et al., 2003; Slobodkina et al., 2009; Handley et al., 2010).

Other highly abundant chemolithotrophic genera that obtain energy via oxidation of reduced chemical species other than iron were detected in our study. These included sulfate-reducing *Thermodesulfovibrio*, nitrate-reducing *Caldithrix*, as well as sulfur-oxidizing *Sulfurimonas* (*Epsilonproteobacteria*), the latter despite the low H_2_S concentrations in Soufrière Bay hydrothermal fluids (1.3 μM H_2_S; Gomez-Saez et al., 2015). In line with these findings, all of these taxa have been previously found at thermally active sites or deep-sea hydrothermal vents (e.g., Henry et al., 1994; Inagaki et al., 2003; Miroshnichenko et al., 2003). The autotrophic bacterial community composition of the Dominica shallow-water vents varied with sediment depth, with a clear dominance of a mixed photo- and chemoautotrophic community in the surface layer and exclusively chemoautotrophic microorganisms in the deeper layers. This is consistent with the findings at another iron-enriched shallow-water hydrothermal systems off Santorini (Greece) (Handley et al., 2010), where similar gradational shift with high abundances of *Mariprofundus, Geothermobacter*, and *Chloroflexi* in the surface layers and *Deltaproteobacteria* (*Desulfuromonadales* and *Desulfobulbus*) in subsurface layers was revealed.

### Linking Lipid Signatures to the Microbial Carbon Metabolism

To further investigate the process of chemoautotrophic carbon fixation, we combined the DNA-based diversity analysis with SIP of lipid biomarkers, which provides information on the metabolic and physiological state of microbial communities in environmental samples (Wegener et al., 2016, and references therein). Increase of ^13^C-incorporation into diagnostic lipids, for instance *10Me-*C_16:0_, points to the activity of iron reducers because this fatty acid has previously been reported to be a specific biomarker for deltaproteobacterium *Geobacter* sp. (Lovley, 1993; Zhang et al., 2003). In our study, highest ^13^C-bicarbonate uptake was determined in the same major fatty acids as previously described for iron reducers (C_16:1ω7_, C_16:0_ and C_18:1ω7_; Zhang et al., 2003), and specifically the fatty acid *10Me-*C_16:0_ might be linked to the deltaproteobacteria genus *Geothermobacter*, which was among the five most abundant genera identified in the whole bacterial community (**Figure 2C**).

Strongest ^13^C-incorporation was measured for fatty acids with a chain length ranging from C_14_ to C_18_, most specifically into *ai*C_15:0_, C_15:0_ and *i*C_16:0_ under dark conditions, which we classified to be potential lipid biomarkers for chemosynthetic bacteria (**Figures 3, 4**). This is consistent with previous literature describing the branched fatty acids *i*C_15:0_ and *ai*C_15:0_ as deriving from sulfate reducing bacteria (SRB; Hinrichs et al., 2000; Niemann and Elvert, 2008; Bühring et al., 2011) or acidophilic microbial communities linked to chemosynthesis (Bühring et al., 2012). Our bacterial analysis would be consistent with the possibility of linking these fatty acids to microbial sulfur cycling (e.g., sulfate-reducing *Thermodesulfovibrio*; **Figure 2C**). Fatty acids showing minor ^13^C-incorporation such as C_18:2_, C_18:1ω9_ and C_18:0_ were not categorized as chemoautotrophic biomarkers, which is consistent with the literature often linking C_18:2_ and C_18:1ω9_ to cyanobacteria and other photosynthetic bacteria (e.g., Gugger et al., 2002; Bühring et al., 2009).

In marine shallow-water hydrothermal systems, chemosynthesis could be enhanced by the increased availability of oxygen as an electron acceptor due to its production by diatoms or cyanobacteria during oxygenic photosynthesis. Nonetheless, our fatty acid results did not support the possibility of active diatoms in the system, as we did not detect long-chain polyunsaturated fatty acids known to be produced by diatoms (Volkman et al., 1989). Furthermore, we detected only minimal tracer incorporation in the light into fatty acids C_18:2_ and C_18:1ω9_ often linked to cyanobacteria (Gugger et al., 2002; Bühring et al., 2009). In contrast, the high relative abundance of sequences belonging to the phylum *Chloroflexi* detected in our study suggests that they could play an important role in the Dominica shallow-water hydrothermal system. *Chloroflexi* function either as heterotrophs or as anoxygenic photoautotrophs. Reports about the fatty acid inventory of *Chloroflexi* vary in the literature with either *ai*C_17:0_, *ai*C_15:0_, *i*C_15:0_, and C_16:0_ (Yamada et al., 2006) or C_16:1ω7_ and C_18:1ω9_ being dominant (Imachi et al., 2014). Interestingly, we classified the former set of fatty acids known to be present in thermo- and mesophilic *Chloroflexi* (Yamada et al., 2006) with a high potential to be chemoautotrophic biomarkers in Dominica (**Figure 4**). In contrast, compounds with ^13^C-label incorporation > 40 pg ^13^C g^-1^ sediment such as C_16:1ω7_ and C_16:0_ have been identified as being widespread among photo- and chemoautotrophic isolates of *Chloroflexi* (Yamada et al., 2006) and were specifically observed in *Pelolinea submarina*, a heterotrophic marine bacterium affiliated with the *Chloroflexi* (Imachi et al., 2014). In our study, the distribution of *Chloroflexi* appeared to be independent of light availability, as their relative abundance did not change with depth, although the incubation of 2 days out of the natural environment might have caused bias in the bacterial distribution results (**Figure 2**). We argue that *Chloroflexi* are unlikely to perform anoxygenic photoautotrophy in the Dominica system, and that most of the fatty acids with high ^13^C-bicarbonate incorporation (i.e., C_16:1ω7_, C_16:0_ and C_18:1ω7_) derive from other chemoautotrophic bacteria. These dominant fatty acids were probably synthesized via the anaerobic pathway of fatty acid biosynthesis, which leads to the production of *ω7* isomers (Alexandrino et al., 2001; Elvert et al., 2003).

Accordingly, the *Chloroflexi* classes identified in our study, i.e., *Anaerolineae, Ardenticatenia, Caldilineae*, and *Dehalococcoidia* have not been reported as photoautotrophs but instead have been considered as heterotrophs (Sekiguchi et al., 2003; Yamada et al., 2006; Kawaichi et al., 2013; Imachi et al., 2014). This would be consistent with the high relative abundance of *Anaerolinea thermophila* in surface and subsurface layers of the iron-rich Santorini shallow-water hydrothermal system, where *Chloroflexi* were also proposed to be heterotrophs, and not contributing to primary production (Handley et al., 2010). However, our incubations were performed for a maximum of 48 h and previous studies have shown that incubation times shorter than one to 2 weeks seem to prevent labeling of heterotrophic organisms due to cross-feeding (Knief et al., 2003). Therefore, our experiments are likely to have primarily targeted autotrophic microorganisms, but co-assimilation of CO_2_ by autotrophs and active members of the heterotrophic community, including thermo- or mesophilic *Chloroflexi*, cannot be fully excluded (e.g., Roslev et al., 2004; Yamada et al., 2006; Wegener et al., 2012; Yakimov et al., 2014; Schubotz et al., 2015).

### Relative Contribution of Chemoautotrophy to Primary Production

In coastal sediments, the rate of dark carbon fixation was generally considered low due to the high competition for electron donors (Jørgensen and Nelson, 2004). However, recent studies concluded that dark carbon fixation by chemoautotrophic bacteria can be a major process in the carbon cycle of coastal sediments (Middelburg, 2011; Boschker et al., 2014; Dyksma et al., 2016). In particular, shallow near-shore sediments can contribute up to 47% to chemoautotrophic carbon fixation in the ocean (Middelburg, 2011). At marine shallow-water hydrothermal systems, chemosynthesis driven by the availability of reduced chemicals is a process that co-occurs with photosynthesis (Tarasov et al., 2005), thereby contributing to primary production. Previous estimates have shown that the proportion of chemosynthesis to total primary production at shallow-water hydrothermal vents can vary between 1 and 50% (Tarasov et al., 2005). Here, we determined that according to our incubations chemoautotrophy could account for of up to 65% of the autotrophic carbon fixation into fatty acids (**Figure 3C**; dashed lines), potentially constituting an important input of newly synthesized organic matter for this coastal ecosystem.

Chemoautotrophy was detected mainly in the surface layer (0–2 cm) and up to 8 cm sediment depth during short time incubations of 6 and 12 h (**Figure 5**). However, longer incubations lead to slower incorporation rates, especially in the deeper layers, most likely due to the limited supply of both reduced substrates, e.g., iron (II), from below and oxidized chemicals, e.g., oxygen or nitrate, from above that are replenished *in situ* by hydrothermal circulation and are required for chemosynthesis. This is supported by the observation that chemosynthesis was initially present at similar depths in our study as in another study focusing on intertidal permeable sediments (e.g., Enoksson and Samuelsson, 1987; Lenk et al., 2011), where oxidants such as oxygen were transported deeper into the sediment by advective processes (Billerbeck et al., 2006). In contrast, in sulfidic marine coastal sediments from the North Sea dominated by diffusion, chemoautotrophy was restricted to the oxygenated top 0.5 cm of the sediment, and below 1 cm depth chemosynthesis could not be measured (Boschker et al., 2014). This supports the critical role of hydrothermal circulation in the permeable sediments of the Dominica shallow-water hydrothermal system in driving chemosynthesis in deeper sediment layers.

Given the relevance of chemosynthesis in the carbon cycle (e.g., Hügler and Sievert, 2011; Middelburg, 2011), its relative importance for primary production should be quantified in more environments where chemosynthetic activity occurs due to geological, biological or anthropogenic processes. To our knowledge, very few studies have quantified rates of chemoautotrophic production in marine coastal environments or brackish lake sediments not influenced by hydrothermal activity (Enoksson and Samuelsson, 1987; Thomsen and Kristensen, 1997; Lenk et al., 2011; Middelburg, 2011; Santoro et al., 2013; Boschker et al., 2014; Dyksma et al., 2016). Therefore, global estimates of chemoautotrophy are currently limited (e.g., Middelburg, 2011) and quantitative approaches such as deployed in the present study are needed to get a better understanding of the relevance of carbon fixation in various marine and terrestrial environments.

## Conclusion

In the present study, we combined SIP of lipid biomarkers with DNA-based bacterial community structure analysis to investigate the relative importance of chemoautotrophy in a light-exposed, iron-enriched marine shallow-water hydrothermal system off Dominica (Lesser Antilles). According to our incubations, we estimated that chemoautotrophy could account for of up to 65% of the autotrophic carbon fixation into fatty acids, potentially constituting an important contribution of newly synthesized organic matter for this coastal ecosystem. Relatively elevated ^13^C-incorporation under dark conditions allowed classification of branched and odd-chain fatty acids *ai*C_15:0_, C_15:0_ and *i*C_16:0_ as potential lipid biomarkers for chemoautotrophic bacteria in the Dominica system. Analysis of the bacterial diversity revealed *Anaerolineae* of the *Chloroflexi* as the most abundant bacterial class. Furthermore, our study identified the Dominica marine shallow-water hydrothermal system as a hotspot for microbes involved in iron cycling (e.g., *Zetaproteobacteria* and *Geothermobacter*), as well as other chemoautotrophic bacteria generally known from deep-sea hydrothermal vents.

## Author Contributions

GG-S and SB designed the research. GG-S, PPR, SS, and SB carried out field sampling. GG-S and PPR performed laboratory work. GG-S analyzed data and wrote the manuscript with help and input from all co-authors.

## Conflict of Interest Statement

The authors declare that the research was conducted in the absence of any commercial or financial relationships that could be construed as a potential conflict of interest.
